# Current State of AIDS-Related Malignant Lymphoma

**DOI:** 10.3390/v17070904

**Published:** 2025-06-26

**Authors:** Seiji Okada, Shotaro Hagiwara, Hirokazu Nagai

**Affiliations:** 1 Division of Hematopoiesis, Joint Research Center for Human Retrovirus Infection, Kumamoto University, Kumamoto 860-0811, Japan; 2 Graduate School of Medical Sciences, Kumamoto University, Kumamoto 860-0811, Japan; 3 Department of Hematology, Tsukuba University Hospital Mito Clinical Education and Training Center, 3-2-7 Miya-Cho, Mito 310-0015, Japan; hagiwara.shotaro@md.tsukuba.ac.jp; 4 Department of Hematology, National Hospital Organization Nagoya Medical Center, 4-1-1 Sannomaru, Naka-ku, Nagoya 460-0001, Japan; hirokazu.nagai@nnh.go.jp

**Keywords:** AIDS, malignant lymphoma, Epstein–Barr virus: EBV, Kaposi sarcoma-associated herpesvirus: KSHV

## Abstract

AIDS-related malignant lymphomas (ARLs) are the lymphomas that develop in association with HIV infection. According to the introduction of combination antiretroviral therapy (cART), the life expectancy of People Living with HIV (PLWH) has markedly improved; however, approximately one-third of PLWH have passed away from the complications of malignancies, even in well-controlled PLWH. HIV itself is not tumorigenic, and most of these tumors are due to co-infection with oncogenic viruses. γ-herpes viruses (Epstein–Barr virus: EBV and Kaposi sarcoma-associated herpesvirus: KSHV) are the most significant risk factors for ARLs. Immunodeficiency, chronic inflammation, accelerated aging, and genetic instability caused by HIV infection, as well as HIV accessory molecules, are thought to promote lymphomagenesis. The prognosis of ARLs is comparable to that of non-HIV cases in the cART era. Intensive chemotherapy with autologous stem cell transplantation is also available for relapsed/refractory ARLs. Since the early stage of HIV infection has no symptoms, significant numbers of HIV-infected individuals have not noticed HIV infection until the onset of AIDS (so-called Ikinari AIDS). Since the ratio of these patients is more than 30% in Japan, hematologists should carefully consider the possibility of HIV infection in cases of lymphoma. Even in an era of cART, ARL remains a critical complication in PLWH, warranting continuous surveillance.

## 1. Introduction

The Joint United Nations Programme on HIV/AIDS (UNAIDS) reported that by the end of 2023, the cumulative number of People Living with HIV (PLWH) was approximately 90 million, and the number of deaths had reached 4.2 million. Currently, the number of PLWH is estimated to be approximately 40 million, with 1.3 million new HIV-infected individuals each year. The cumulative number of registered PLWH in Japan is 35,381, approximately 90% of whom are male. The prognosis for PLWH used to be extremely poor and was considered a “deadly disease,” however; with the introduction of combination antiretroviral therapy (cART), which combines several anti-HIV drugs with different mechanisms of action, the number of deaths due to opportunistic infections has decreased, and HIV/AIDS is now considered a controllable chronic infectious disease. However, due to factors such as the aging of HIV-infected patients, co-infection with tumor viruses, chronic immunodeficiency and inflammation, and lifestyle, approximately one in three HIV-1-infected patients now die from malignant tumors [1]. Non-Hodgkin’s lymphoma and cerebral lymphoma are AIDS indicator diseases, and since AIDS-related malignant lymphoma (AIDS lymphoma) is associated with 5–20% of HIV-infected patients and is the leading cause of death in countries where cART is widespread, it is becoming the most critical factor defining long-term prognosis of HIV-1-infected patients. Currently, approximately 30% of newly HIV-infected patients are first diagnosed with HIV infection when they develop AIDS, which is referred to as “Sudden onset of AIDS”. It should be noted that some cases present with malignant lymphoma as the initial symptom, which may lead to a visit to a hematologist or neurosurgeon.

## 2. Risk Factors in AIDS-Related Malignant Lymphoma

HIV-infected patients are more frequently associated with malignant tumors than healthy individuals [2]. Still, HIV itself is not tumorigenic, and most of the malignant tumors are caused by overlapping infections with oncogenic viruses (Table 1). Especially, γ-herpes viruses—Epstein–Barr virus (EBV, human herpesvirus 4; HHV-4) and Kaposi sarcoma-associated herpesvirus (KSHV; human herpesvirus 8; HHV8) infection—are the most critical risk factors for AIDS lymphoma [3], and it is thought that immunodeficiency, chronic inflammation, accelerated aging, and genetic instability caused by HIV infection are thought to promote the development of lymphoma [4,5]. In addition, HIV component proteins such as Nef, Tat, and p17 are known to activate various pathways of B cells [6], including the BCR (B cell receptor) pathway [6,7]. It has also been suggested that HIV infection induces epithelial to mesenchymal transition (EMT) by decreasing Hsa-miR-200c-3p and increasing ZEB1/ZBE2, which is also involved in cancer invasion and metastasis, and that increased miRNA-21 activates B cells [5]; however, the extent to which miRNA-21 is involved in the development of lymphoma remains to be determined.

### 2.1. Epstein–Barr Virus (EBV) Infection and AIDS Lymphoma

AIDS lymphomas are mostly B-cell lymphomas, presenting at various stages of B-cell differentiation (Table 2) [8,9]. More than 90% of the population worldwide is infected with EBV and is latently infected [10], and many cases of AIDS lymphoma are positive for EBV, but the form of the latent infection varies by disease type (Table 3). Diffuse large B cell lymphoma (DLBCL) is classified into the centroblastic and immunoblastic types. EBV infection is observed in 90% of immunoblastic lymphomas (Table 2) [11], while EBV infection is observed in 20–30% of immunoblastic lymphomas. Additionally, the latent EBV infection gene LMP-1 is detected in approximately 90% of cases of immunoblastic lymphoma. In contrast, it is rarely seen in the germinal center blastic type, suggesting that EBV infection plays a significant role in the development of AIDS lymphoma. Burkitt lymphoma (BL), which has been increasing in recent years [12], typically presents with type I latent infection and develops after CD4 recovery with cART treatment. In contrast, Hodgkin lymphoma presents with type II latent infection and develops during the period of immunodeficiency recovery after cART induction. Primary effusion lymphoma (PEL) and plasmablastic lymphoma (PBL) present in an immunocompromised state, both of them with type I latent infection, although the relationship of EBV to the pathogenesis is unknown.

### 2.2. HHV8/KSHV Infection and AIDS Lymphoma

HHV8/KSHV belongs to the γ-herpes virus and was first identified as the causative virus of Kaposi’s sarcoma in 1994 [13], PEL in HIV-infected individuals [14], and the increasing frequency of Castleman’s disease in HIV-infected individuals in recent years. In addition, there have been cases of Castleman’s disease progressing to HHV-8-positive DLBCL, not otherwise specified (NOS) [15,16] (Table 4).

### 2.3. Hepatitis Virus Infection and AIDS Lymphoma

Hepatitis B (HBV) and Hepatitis C (HCV) have been shown to be associated with the development of lymphoma, and many cases of PLWH are co-infected with HBV/HCV because they share the risk factor, especially in HIV-infected patients infected with blood products or drug users [17]. HBV/HCV infection is a risk factor not only for hepatocellular carcinoma but also malignant lymphoma [18], and HCV co-infection is shown to increase the frequency of non-Hodgkin lymphoma in HIV-infected patients [19]. On the other hand, there are reports that the frequency of lymphoma development is not different from that reported to increase with HBV co-infection [20]. Thus, HBV/HCV may be playing a role in the increased risk for lymphoma; however, the role of HBV/HCV in the development of AIDS lymphoma is unknown.

## 3. Malignant Lymphomas Associated with HIV-1 Infection

### 3.1. Non-Hodgkin Lymphoma (NHL)

Non-Hodgkin lymphoma (NHL) is one of the AIDS indicator diseases and is currently one of the leading causes of death among AIDS patients in countries where cART is widespread, including Japan [21,22]. Most of them are B-cell lymphomas, with diffuse large B-cell lymphoma accounting for about 50%, followed by Burkitt’s lymphoma at about 30%. The results of treatment with chemotherapy combined with cART have been improving in both cases. Primary effusion lymphoma and plasmablastic lymphoma occur mainly in untreated HIV-infected patients and have a poor prognosis [8].

#### 3.1.1. Diffuse Large B Cell Lymphoma (DLBCL)

DLBCL is the most common form of ARL. Prior to the introduction of cART, immunoblastic DLBCL was more common in immunocompromised patients due to infection with the germinal center blasts, but since the introduction of cART, the proportion of germinal center blasts has increased. The pathogenesis of DLBCL without involvement of EBV infection is unknown. In patients without significant opportunistic infection, R-CHOP or R-EPOCH therapy is the treatment of choice, as in non-AIDS patients, while continuing cART [23], and treatment efficacy comparable to that in non-AIDS patients has been achieved [24]. In refractory or relapsed cases, intense chemotherapy with autologous peripheral blood stem cell transplantation is effective [25].

#### 3.1.2. Burkitt Lymphoma (BL)

The number of BL cases has been increasing since the introduction of cART [4,12]. Around 30–50% of cases are associated with EBV infection, and the pathogenesis differs from that of sporadic and endemic Burkitt lymphoma (Table 5), as the c-myc translocation site is different [26,27]. It is often associated with cases in which CD4 counts have been restored by cART [28,29]. It is hard to distinguish BL and DLBCL by H&E staining because AIDS-associated BL shows diffuse growth of rather large tumor cells that do not show the typical Starry sky by inappropriate inflammation due to HIV infection [30] (Table 6). Immunostaining and c-myc rearrangement are needed for diagnosis. BL needs more intensive treatments (Hyper CVAD and R-CODOX-M/IVAC), and the prognosis is getting better after the cART era [12,31].

#### 3.1.3. Primary Effusion Lymphoma (PEL)

PEL is a rare lymphoma that grows in body fluids without forming a mass, is resistant to chemotherapy, and has a poor prognosis [32]. It is caused by HHV-8 infection, and many cases show EBV coinfection, but the role and significance of EBV infection is unknown. In recent years, extra cavitary PEL forming solid tumors have been reported [33]. Proof of HHV-8 infection by LANA immunostaining or other methods is essential for diagnosis. Even today, when the prognosis of other HIV-associated malignant lymphomas has improved, the prognosis of PEL with conventional chemotherapy is still very poor [34]. Immunotherapy and molecular targeted therapy, such as antibody therapy and CAR-T therapy, are expected to show promising results [35].

#### 3.1.4. Plasmablastic Lymphoma (PBL)

Plasmablastic lymphoma (PBL) is a rare, CD20-negative, and aggressive B-cell lymphoma characterized by plasmablastic morphology [36,37]. Most cases are in males with low CD4 counts, and EBV infection is involved in 100% of cases. It is often shown in the oral cavity, and biopsy is necessary when oral ulcers develop in HIV-infected patients [6,37]. In recent years, PBL originating from the gastrointestinal tract and other organs, as well as systemic PBL, have been increasing. The incidence of PBL in Japan has also been increasing, and caution should be exercised in its diagnosis [38]. The prognosis is poor, but aggressive chemotherapy, including autologous stem cell transplantation, is successful [39,40].

### 3.2. Primary Central Nervous System Lymphoma (PCNSL)

PCNSL is an intracranial lymphoma and one of the AIDS indicator diseases. The histological type is predominantly DLBCL immunoblastoid, and it often occurs in patients with low CD4 counts (<50/μL). EBV infection is observed in almost all cases. Since some patients present with PCNSL as the first manifestation of AIDS (sudden onset of AIDS), caution should be exercised in diagnosis. It is difficult to distinguish EBV infection from Toxoplasma infection by CT; however, FDG-PET and confirmation of EBV in spinal fluid are useful for diagnosis [41,42]. Although treatment requires aggressive chemotherapy such as high-dose MTX (Methotrexate), there have been reports of PCNSL cured by improving immunodeficiency with cART alone. Whole brain irradiation is considered adequate, but late-onset leukoencephalopathy is often a problem as a sequela [43]. In a retrospective study in Japan, whole-brain irradiation was effective in patients with a good performance status (PS) [44].

### 3.3. Hodgkin Lymphoma (HL)

Although Hodgkin lymphoma is not an AIDS indicator disease, its incidence of HL has increased after the introduction of cART [45,46]. An inflammatory response is considered to play a crucial role in the development of HL, and its incidence is low in immunodeficient patients [47]. Characteristics of HL in HIV-1-infected patients include a high rate of EBV infection (80–100%), dominance of mixed cellularity (MC) and lymphocyte depletion (LD) types, advanced stage at the time of diagnosis, high frequency of extranodal involvement (especially bone marrow infiltration), and poor performance status [46,48]. HL in PLWH is increasing in Japan and cART-accessible countries, especially during the CD4 recovery phase; however, the prognosis is relatively good [49].

## 4. Advances in the Treatment of AIDS-Related Malignant Lymphoma

Before the cART era, treatment of ARL was complex and had an extremely poor prognosis due to the poor performance status at diagnosis and complications of severe opportunistic infection. Although the introduction of cART has improved the prognosis of ARL, concomitant use of anti-tumor drugs, anti-HIV drugs, and anti-infective agents during treatment requires paying attention to drug interactions. In particular, since protease inhibitors inhibit CYP3A4, it caused severe side effects. Recently, according to the introduction of integrase inhibitors, drug interactions have become less of a problem, and the outcome of treatment and prognosis of patients have improved to a level comparable to that of non-HIV cases. In addition, the recent approval of long-acting injectable drugs, such as cabotegravir and rilpivirine, is expected to further enhance therapeutic efficacy by eliminating the need for anti-HIV drugs during lymphoma treatment. On the other hand, the number of cases of complications of other malignancies after treatment for AIDS-related malignant lymphoma is increasing, and follow-up is necessary [50].

## 5. Conclusions

The incidence of non-Hodgkin lymphoma in HIV-infected patients is 23 times higher than that in the general population, and approximately 7% of all non-Hodgkin lymphoma cases in the world are in PLWH. After the introduction of cART, although the number of opportunistic infections has significantly decreased, malignant lymphoma has become the most critical complication in determining the prognosis of PLWH. It is important to note that lymphoma may develop as a first manifestation of AIDS, and that lymphoma may develop when AIDS is stabilized by treatment. It is hoped that further clarification of the pathogenesis of ARL will lead to the establishment of preventive measures.

## Figures and Tables

**Table 1:** HIV infection and cancers.

HIV Relation	Malignancies		Virus	Feature
AIDS-defining cancer	Kaposi’s sarcoma		HHV8/KSHV	Low CD4
	Lymphoma	DLBCL	EBV (50%)	Low CD4
		Burkitt	EBV (30–40%)	Moderate CD4
		PEL	HHV8/KSHV	Low CD4
		PBL	EBV (100%)	Low CD4
	PCNSL		EBV (100%)	Low CD4
Non-AIDS- defining cancer	Invasive Cervical Cancer		HPV	Not related CD4
	Anal canal /Oral /Laryngeal cancers		HPV	Not related CD4
	Hodgkin Lymphoma		EBV	Moderate CD4
	Lung cancer		No virus	Smoking
	Liver Cancer (Hepatocellular carcinoma)		HBV/HCV	

HHV8/KSHV: human herpesvirus 8/Kaposi sarcoma-associated herpesvirus, EBV: Epstein–Barr virus, HPV: human papillomavirus, HBV/HCV: Hepatitis B virus/Hepatitis C virus.

**Table 2:** Classification of AIDS-related lymphoma by B cell differentiation stages.

	Germinal Center			Post−Germinal Center			
Type	Germinal Center			Activated B	Plasmacytoid		
Type of Lymphoma	Burkitt	Hodgkin	DLBCL		HHV8(+)−DLBCL	PEL	PBL
			Germinal Center	Immunoblastic			
EBV (+)	30–50%	90–100%	30–40%	90–100%	(0%)	90%	100%
HHV8	−	−	−	−	+	+	−
MUM1 /IRF4	−	−	−	+	+	+	+
CD20	+	+	+	+	+/−	−	−
CD10	+		+	−	−	−	−
CD138	−	−	−	−/+	+/−	+/−	+
BCL6	+		+	−	−	−	−
Immunodeficiency	Mild			Severe			
CD4 numbers	Moderate			Low			
Prognosis	Improved in cART era				Poor prognosis		

**Table 3:** AIDS-related lymphoma and latency of EBV.

Latency Type	EBV Gene Expression	EBV-Related Lymphoma	Other Transforming Alteration	Immunogenicity	Immune State
Latency 0		Healthy		−	Normal
Latency I	EBNA1	BL	c-myc ±p53	±	
		PEL	±BCL-6 HHV8		
		PBL			
Latency II	EBNA1, LMP1	HD	±IκBα	+	Mildly suppressed
Latency III	EBNA1, EBNA2 EBNA3s LMP1, LMP2	DLBCL		++	Severely suppressed

**Table 4:** KSHV/HHV-8-associated diseases.

Diseases and Disorders
Lymphoma
Primary effusion lymphoma, classic, and extracavitary variants
HHV8-positive diffuse large B-cell lymphoma, NOS
Lymphoproliferative diseases
HHV8-positive germinotropic lymphoproliferative disorder (GLPD)
HHV8-positive multicentric Castleman’s disease
Other HHV8-related lymphoproliferations
HHV8-positive reactive lymphoid hyperplasia
Plasmablastic proliferation of the splenic red pulp
KSHV/HHV8 inflammatory cytokine syndrome (KICS)
Sarcoma
Kaposi’s sarcoma

**Table 5:** Etiology and characterization of Burkitt lymphoma (BL).

	Endemic BL	Sporadic BL	AIDS-Associated BL
Geographic Distribution	Equatorial Africa Papua New Guinea	Worldwide (Europe and America)	Worldwide PLWH
Age	Children (median: 6–9 yr)	Adult Children	Adult (median: 40–45 yr)
Incidence	5–10/10^5^	0.01/10^5^	6/1000 PLWH
Associated with EBV	>90%	<20% (higher incidence among elder adults)	30–40%
Site	Extranodal, jaw/orbit	Nodal, Extranodal (abdomen, BM, etc)	Nodal, Extranodal (GI-tract, BM, liver, etc.)
Bone marrow (BM) involvement	10%	30%	30–60%
c-myc translocation	Yes	Yes	Yes
c-myc break point	5′	5′, exon1, intron1	exon1, intron1
IgH break point	VDJ region Switch region	VDJ region Switch region	Switch region
Somatic hypermutation	+++	+	+++
Related factor	EBV, malaria	Unknown	HIV infection

**Table 6:** Comparison of AIDS-related DLBCL and BL.

		DLBCL	BL
Incidence among ARL		50%	30%
Malignancy		Aggressive	Highly Aggressive
Progression		Rapid	Very rapid
Treatment		R-CHOP, R-EPOCH	Hyper CVAD, R-CODOX-M/IVAC
Characteristics	Tumor cell size	Large cell size	Intermediate-large cell
	Starry sky	Negative	Frequently negative
	c-myc translocation	Negative (occasionally positive)	Positive
	MIB-1 (Ku-67)	<90	>90
Prognosis		Poor	Poor, rarely relapse after complete remission

## Abbreviations

The following abbreviations are used in this manuscript:

AIDS	Acquired immune deficiency syndrome
HIV	Human immunodeficiency virus
cART	Combination antiretroviral therapy
PLWH	People Living with HIV
EBV	Epstein–Barr virus
HHV-8/KSHV	Human herpes virus-8/Kaposi sarcoma-associated herpesvirus
DLBCL	Diffuse large B cell lymphoma
PEL	Primary effusion lymphoma
PBL	Plasmablastic lymphoma
NHL	Non-Hodgkin lymphoma
PCNSL	Primary central nervous system lymphoma
